# Low-density lipoprotein-cholesterol lowering effect of a nutraceutical regimen with or without ezetimibe in hypercholesterolaemic patients with statin intolerance

**DOI:** 10.3389/fcvm.2022.1060252

**Published:** 2022-11-25

**Authors:** Natalie C. Ward, Christopher M. Reid, Gerald F. Watts

**Affiliations:** ^1^Dobney Hypertension Centre, Medical School, University of Western Australia, Perth, WA, Australia; ^2^School of Population Health, Curtin University, Perth, WA, Australia; ^3^Medical School, University of Western Australia, Perth, WA, Australia; ^4^Lipid Disorders Clinic, Department of Cardiology, Royal Perth Hospital, Perth, WA, Australia

**Keywords:** statin intolerance, nutraceuticals, ezetimibe, low-density lipoprotein (LDL), lipids

## Abstract

**Background:**

Statins are the most widely prescribed medication to lower low-density lipoprotein cholesterol (LDL-c). However, a significant portion of patients are unable to tolerate them due to side effects, most commonly muscle related. Nutraceuticals, natural plant derivatives with lipid-lowering properties, may provide an alternative to lower LDL-c in these patients.

**Aims:**

To investigate whether a nutraceutical regimen, either alone or in combination with ezetimibe, can lower LDL-c in patients with hypercholesterolemia who are intolerant to statins.

**Methods:**

Participants were recruited into a double-blind, randomized, placebo-controlled intervention study. Treatments were (i) placebo, (ii) nutraceutical (500 mg berberine, 200 mg red yeast rice (RYR), 2 g plant sterols)/daily, (iii) ezetimibe (10 mg)/daily, or (iv) the combination of nutraceutical and ezetimibe/daily. At baseline and week 8, all participants provide a fasting blood sample for assessment of lipid profile and safety bloods.

**Results:**

Fifty participants were randomized, with 44 completing the treatment period. Following adjustment for baseline levels and compared with placebo, LDL-c was significantly reduced (all *p* < 0.0001) with ezetimibe (−1.02 mmol/L), nutraceutical (−1.15 mmol/L) and the nutraceutical and ezetimibe combination (−1.92 mmol/L). Non-HDL cholesterol was significantly reduced (all *p* < 0.0001) with ezetimibe (−1.29 mmol/L), nutraceutical (−1.37 mmol/L) and the nutraceutical and ezetimibe combination (−2.18 mmol/L). Remnant cholesterol and triglycerides was significantly reduced with the nutraceutical and ezetimibe combination (*p* = 0.018).

**Conclusion:**

A nutraceutical regimen (berberine, RYR and plant sterols) and ezetimibe independently and additively lower LDL-c in patients with hypercholesterolemia who are intolerant to statins.

## Introduction

Cardiovascular disease (CVD) is the leading cause of death. There is significant evidence to support lowering low-density lipoprotein cholesterol (LDL-c) to reduce atherosclerotic cardiovascular disease (ASCVD) (1). Meta-analysis highlights for every 1 mmol/L LDL-c reduction there is a significant 22% relative risk reduction in major vascular and coronary events (2). Statins are the most widely prescribed and evidence-based drugs for lowering LDL-c and reducing cardiovascular events (3). International guidelines recommend the use of statin therapy to reduce risk in a range of patient populations (clinical ASCVD, diabetes mellitus and hyperlipidemia), where the greater the LDL-c reduction (by >50%.), the greater the subsequent risk reduction (4, 5).

Statins competitively block the active site of HMG-CoA reductase, reducing hepatic cholesterol synthesis and increasing cell surface LDL receptor expression (6). This increases clearance of LDL-c from the bloodstream and reduces circulating levels by 20–55% (6). Combination therapy with ezetimibe has additional beneficial effects on both LDL-c and cardiovascular outcomes (7, 8). Statin toxicity or intolerance is most commonly reported as statin-associated muscle symptoms (SAMS), presenting as myalgia, myopathy, myositis with elevated CK, or at its most severe, rhabdomyolysis, with some people reporting additional joint and abdominal pain (9–11). Ezetimibe works by blocking the NPC1L1 transporter, inhibiting the absorption of cholesterol in the intestine, upregulating the LDL receptor and thereby lowering LDL-c. As demonstrated by the IMPROVE-IT study, it has incremental effects in lowering LDL-c and cardiovascular events in high risk populations when added to statin therapy (7, 8). Nutraceuticals are plant or food-based compounds or derivatives that provide health benefits (12, 13), including improving lipid profiles (14–16). Their use as adjuncts to standard pharmacotherapy is recommended by most international guidelines and in addition to their efficacy, their use may also be advantageous in terms of safety and tolerance, particularly in patients with documented SAMS (15). Berberine, red yeast rice (RYR), and plant sterols, have all been demonstrated to modestly reduce LDL-c *via* distinct yet complimentary mechanisms (16).

The aim of this pilot study was to investigate the efficacy of a nutraceutical regimen, either alone or in combination with ezetimibe, on LDL-c in hypercholesterolaemic patients with documented statin intolerance who are not currently achieving lipid targets. We hypothesized that the nutraceutical regimen, consisting of berberine, RYR and plant sterols, and ezetimibe would independently and additively lower plasma concentration of LDL-c in these patients.

## Study design and methods

### Participants

Participants with documented statin intolerance and not currently achieving ideal lipid goals (fasting LDL-c > 1.8 mmol/L and < 7 mmol/L) were recruited from the Lipid Disorders Clinic, Department of Cardiology, Royal Perth Hospital. Statin intolerance was defined as an inability to tolerate two or more statins due to side effects (including myalgia) that started or increased during statin therapy and resolved on cessation, or severe intolerance to one statin at multiple doses, resulting in refusal to try additional statins. Participants who were taking ezetimibe or other cholesterol-lowering medication at screening (*n* = 20) underwent a 4-week washout prior to randomization. Exclusion criteria included recent acute coronary syndrome (<3 months), abnormal plasma biochemistry at screening suggesting an underlying cause of statin intolerance (e.g., abnormal thyroid function, concomitant medication use, hepatic or renal impairment), elevated creatine kinase (two times the upper limit of normal), pregnant or lactating women, < 30 or > 80 years of age, history of necrotizing auto-immune myositis or autoantibodies against HMG-CoA reductase, or inability to provide consent. The study was carried out in accordance with Good Clinical Practice and was approved by Royal Perth Hospital Human Research Ethics Committee (RGS0891). The trial was registered at www.anzctr.org.au as ACTRN12618000660280.

### Study design and interventions

A randomized, double-blind, placebo-controlled study was carried out between May 2019 and January 2022. Participants underwent screening to assess suitability to take part in the study, which included assessment of their previous statin intolerance, a fasting blood sample (lipid profile, blood glucose, HbA1c, serum creatinine, renal function, liver function, vitamin D, full blood picture and thyroid function), measurement of height and weight, clinic BP, medical history, and medical assessment. All had received previous medical advice at first review in clinic on a fat-modified diet as well as the need to achieve a desirable body weight with diet and exercise. Following screening, participants were randomly allocated to a treatment group by computer generated random numbers. Treatment groups were (i) nutraceutical placebo + ezetimibe placebo; (ii) nutraceutical active + ezetimibe placebo; (iii) nutraceutical placebo + ezetimibe active; or (iv) nutraceutical active + ezetimibe active. The treatment period was 8 weeks.

Study treatments were prepared by Optima Ovest (Perth, Australia), a GMP licensed company and dispensed through Oxford Compounding Pharmacy in compliance with Therapeutic Goods Administration (TGA) requirements. The nutraceutical treatment regimen contained 500 mg berberine (berberine chloride), 200 mg RYR (equivalent to 3 mg monacolin K) and 2 g of plant sterols (vegetable oil plant sterol esters). Ezetimibe was given as a 10 mg dose, and the placebo contained avicel (microcrystalline cellulose). Active agents were encapsulated to ensure all medications were identical in appearance. Tablets were taken daily with food and compliance monitored through tablet count.

### Lipid and lipoprotein analysis

Fasting plasma lipid profiles [total cholesterol, LDL-c, triglycerides, high-density lipoprotein cholesterol (HDL-c), non-HDL-c and remnant cholesterol (REM-c)] were assessed at weeks 0, 4 and 8. Total cholesterol, triglycerides and HDL-c were analyzed using enzymatic colorimetric assays (Abbot Architect c16000 Analyser) by PathWest Laboratories with reagents traceable to the National Reference System. LDL-c was calculated using the Friedewald equation, non-HDL-c was calculated by subtracting HDL-c from total cholesterol, and REM-c was calculated by subtracting HDL-c and LDL-c from total cholesterol.

### Adverse events and participant reported quality of life outcomes

Safety biochemistry (creatine kinase, liver function, renal function, thyroid function and full blood picture) was performed at weeks 0 and 8 by PathWest Laboratories. All results flagged as being outside normal ranges were reviewed by the study clinician. All participants completed EQUAL EQ-5D quality of life questionnaire at weeks 0 and 8. In addition, regular contact was maintained with all participants to document any side effects and/or adverse events. Patient reported myalgia during the study treatment period was documented to include location and self-reported pain level, with review by the study clinician.

### Statistical analyses

This was a pilot study investigating the safety and efficacy of nutraceuticals with or without ezetimibe in patients intolerant to statins. The primary outcome was reduction in LDL-c. Secondary outcomes were acceptability of the study treatments, including development of side effects and self-reported quality of life. Power calculations were based on 80 participants required to see a 0.8 mmol/L reduction in LDL-c with either the nutraceutical or ezetimibe intervention. Interim analysis of the results revealed significant improvements in LDL-c and as a result, the study was concluded early. Analysis of the results was carried out using the Statistical Packages for the Social Sciences (SPSS, Version 26). Analysis was carried out both as per protocol (all patients who completed the full 8-week treatment period) and on an intention-to-treat basis (patients who completed part of the 8-week treatment period), with results presented as mean ± SEM or percentage change from baseline. Analysis was carried out using a maximum-likelihood random effect mixed regression model for main effects, with intergroup comparisons performed using univariate analysis of variance (ANOVA) with Bonferroni post-hoc analysis to account for multiple comparisons. Categorical variables were analyzed using Chi-square. Statistical significance was considered if *p* < 0.05.

## Results

### Participant characteristics

Ninety-nine participants from the Lipid Disorders Clinic were identified and screened. Of these, 49 were excluded or not interested in taking part, with the remaining 50 randomized to treatment. Previous documented statin intolerance revealed 3 participants had previously tried only 1 statin at multiple doses and experienced severe myalgia resulting in refusal to try additional statins. The remaining 47 participants had all tried = 2 statins at one or more doses and experienced myalgia. Additional self-reported side effects on statins included joint aches (*n* = 4), gastrointestinal issues (dyspepsia, bloating, nausea, *n* = 4) and/or neurological changes (brain fog, *n* = 7). The treatment groups were well matched at baseline, with no significant differences for age, gender, BMI, blood pressure or lipid profile. As expected, all participants had elevated total cholesterol, LDL-c and non-HDL-c (Table 1). Four (8%) patients had probable phenotypic familial hypercholesterolemia (FH, fasting LDL-c > 4.9 mmol/L and a Dutch Lipid Score = 5) and 14 (28%) patients had combined hyperlipidemia (fasting LDL-c > 4 mmol/L, triglycerides > 2 mmol/L, obesity). Twelve (24%) patients had evidence of coronary artery disease, with 9 having elevated coronary artery calcium score (CACS ranging from 60 to 395), with 2 having undergone previous percutaneous coronary intervention and 1 a coronary artery bypass graft. In addition, 16 (32%) had hypertension, with 15 on anti-hypertensive therapy and 3 (6%) patients had type 2 diabetes mellitus with all receiving treatment. Fourteen (28%) patients were on aspirin and 1 (2%) was on anti-platelet therapy (Table 1). Using the Australian Absolute Cardiovascular Risk Score calculator (excluding patients with probable FH and previous coronary revascularization), the average 5-year risk score at baseline was 11, placing the cohort at moderate-to-high risk (10–15%). Only 1 patient had had a previous coronary event (CABG). None of the patients met the Australian criteria for Pharmaceutical Benefits Schedule re-imbursement of a PCSK9 inhibitor.

**TABLE 1:** Baseline characteristics of study participants.

	Placebo	Ezetimibe	Nutraceutical	Combination	*P-*value
Number	13	13	8	10	
Sex (male/female)	6/7	8/5	4/4	3/7	0.516
Age (years)	61.8 ± 2.9	61.8 ± 2.0	62.2 ± 3.8	62.8 ± 3.3	0.994
BMI (kg/m^2^)	27.6 ± 1.0	27.8 ± 1.1	30.3 ± 1.4	29.5 ± 1.1	0.311
BMI > 28 kg/m^2^	4	6	5	6	0.515
Blood pressure (mmHg)	134/75	135/78	136/70	136/74	0.288
Hypertension	4	5	5	2	0.293
On anti-hypertensive medication	4	4	5	2	0.274
Total cholesterol (mmol/L)	7.15 ± 0.38	7.12 ± 0.24	6.66 ± .033	7.18 ± 0.33	0.672
LDL-c (mmol/L)	4.86 ± 0.32	4.89 ± 0.25	4.41 ± 0.24	5.01 ± 0.31	0.475
Non-HDL-c (mmol/L)	5.77 ± 0.40	5.75 ± 0.27	5.36 ± 0.38	5.94 ± 0.34	0.769
REM-c (mmol/L)	0.91 ± 0.15	0.85 ± 0.13	0.93 ± 0.13	0.86 ± 0.08	0.975
Triglycerides (mmol/L)	2.08 ± 0.36	1.87 ± 0.28	2.19 ± 0.27	1.77 ± 0.15	0.745
HDL-c (mmol/L)	1.39 ± 0.11	1.38 ± 0.08	1.24 ± 0.13	1.37 ± 0.10	0.756
Common hypercholesterolemia	7	9	3	7	0.646
Combined hyperlipidemia	4	3	5	2	0.643
Familial hypercholesterolemia	2	1	0	1	0.691
Previous CAD	4	4	2	2	0.930
Elevated CAC score	4	4	1	0	0.341
PCI	0	0	1	1	0.341
CABG	0	0	0	1	0.341
Atrial fibrillation	2	0	0	1	0.362
Aspirin (%)	5	4	2	3	0.928
Anti-platelet therapy (%)	0	0	1	0	0.203
Type 2 diabetes mellitus	1	1	1	0	0.789
Medication for diabetes	1	1	1	0	0.760

Mean ± SEM. CACS, previously documented coronary artery calcium score, 60–395. BMI, body mass index; CABG, coronary artery bypass graft; CAC, coronary artery calcium; CAD, coronary artery disease; HDL-c, high-density lipoprotein cholesterol; LDL-c, low-density lipoprotein cholesterol; PCI, percutaneous coronary intervention; REM-c, remnant cholesterol.

### Effects of study treatments on lipid profiles

**FIGURE 1:**
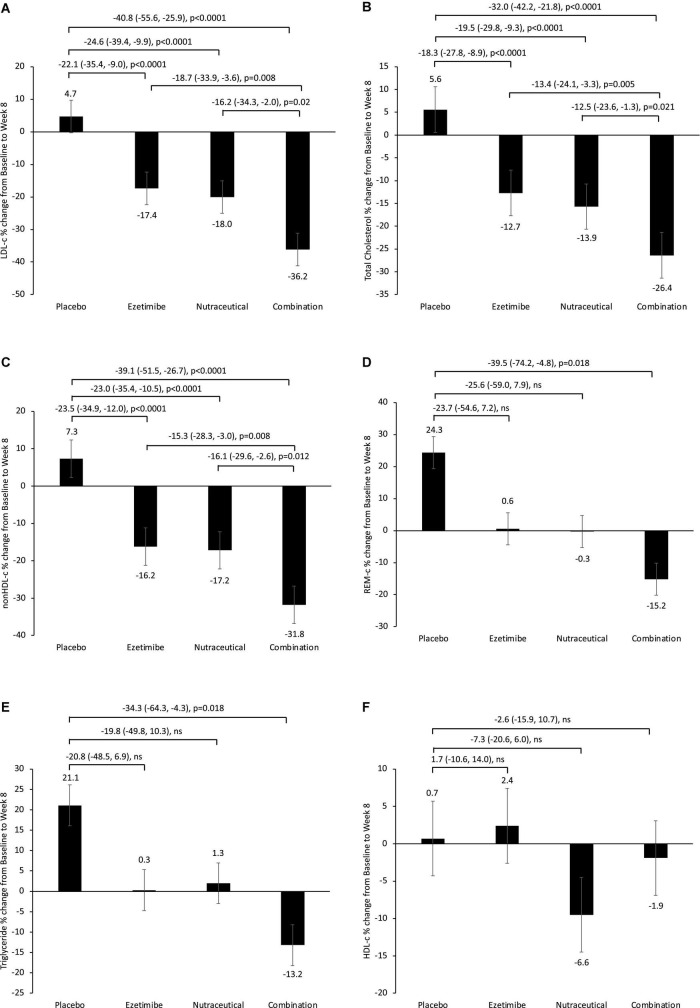
Percentage change from baseline in fasting plasma **(A)** LDL-c, **(B)** total cholesterol, **(C)** non-HDL-c, **(D)** REM-c, **(E)** triglycerides and **(F)** HDL-c, following 8-week treatment with placebo, ezetimibe, nutraceutical regimen, or combination. Intergroup comparisons *via* univariate ANOVA with Bonferroni post-host comparisons.

Analysis of main effects revealed no significant interaction between the ezetimibe and nutraceutical treatments (Table 2). There were significant main effect reductions in LDL-c, total cholesterol and non-HDL-c with the nutraceuticals compared with placebo, as well as with ezetimibe compared with placebo, but no significant effect of either intervention on triglycerides, HDL-c or REM-c (Table 2). Comparative analysis of all four treatment groups individually revealed significant reductions in LDL-c of 22, 25, and 41% with the ezetimibe, nutraceutical and combination therapies, respectively, compared with placebo (Figure 1A). There were also significant reductions in total cholesterol and non-HDL-c with the ezetimibe, nutraceutical and combination therapies compared with placebo (Figures 1B,C). REM-c and triglycerides were significantly lower with the combination therapy compared with placebo (Figures 1D,E), while HDL-c was not altered by any of the treatments (Figure 1F). Within the whole cohort, only one patient attained an LDL-c of < 2.5 mmol/L at week 8 and this was with the nutraceutical and ezetimibe combination therapy.

**TABLE 2:** Effect of the interventions on plasma total cholesterol, LDL-c, non-HDL-c, REM-c, triglycerides, and HDL-c over the 8-week treatment period.

	Placebo		Ezetimibe		Nutraceutical		Combination		Main effects *P-*value		Interactive *P-*value
	Week 0	Week 8	Week 0	Week 8	Week 0	Week 8	Week 0	Week 8	Ezetimibe	Nutraceutical	
Total cholesterol (mmol/L)	7.15 ± 0.38	7.49 ± 0.36	7.12 ± 0.24	6.24 ± 0.23	6.66 ± 0.33	5.56 ± 0.29	7.18 ± 0.33	5.27 ± 0.35	<0.0001	<0.0001	0.275
LDL-c (mmol/L)	4.86 ± 0.32	5.01 ± 0.31	4.89 ± 0.25	4.01 ± 0.20	4.41 ± 0.24	3.54 ± 0.16	5.01 ± 0.31	3.32 ± 0.13	<0.0001	<0.0001	0.631
Non-HDL-c (mmol/L)	5.77 ± 0.40	6.11 ± 0.39	5.75 ± 0.27	4.81 ± 0.26	5.36 ± 0.38	4.41 ± 0.27	5.94 ± 0.34	3.98 ± 0.28	<0.0001	<0.0001	0.267
REM-c (mmol/L)	0.91 ± 0.15	1.10 ± 0.20	0.85 ± 0.13	0.80 ± 0.08	0.93 ± 0.13	0.86 ± 0.09	0.86 ± 0.08	0.66 ± 0.07	0.076	0.129	0.469
Triglycerides (mmol/L)	2.08 ± 0.36	2.50 ± 0.31	1.87 ± 0.28	1.74 ± 0.18	2.19 ± 0.27	1.93 ± 0.18	1.77 ± 0.15	1.44 ± 0.15	0.096	0.191	0.435
HDL-c (mmol/L)	1.39 ± 0.11	1.38 ± 0.09	1.38 ± 0.08	1.43 ± 0.07	1.24 ± 0.13	1.15 ± 0.16	1.37 ± 0.10	1.29 ± 0.13	0.107	0.053	0.912

Mean ± SEM. HDL-c, high-density lipoprotein cholesterol; LDL-c, low-density lipoprotein cholesterol; REM-c, remnant cholesterol.

### Side effects, adverse events and self-reported quality of life

Six participants withdrew from the study within the first week following randomization (1 in the ezetimibe, 3 in the nutraceutical and 2 in the combination group) due to side effects (gastrointestinal, myalgia and joint aches) and were not included in the analysis. Three participants were unable to complete the full 8-week treatment period (1 in ezetimibe, 1 in nutraceutical and 1 in the combination group) due to unrelated events (admission for coronary artery bypass graft, flare up of ulcerative colitis and development of a rash following a spider bite) and were included in the intention-to-treat analysis. Minor gastrointestinal and myalgia side effects were reported equally across all four treatment groups. Serum creatine kinase levels, along with other safety blood parameters (liver and renal function) were not significantly different at weeks 0 and 8, nor were there any significant changes with any of the study treatments (Table 3). Analysis of the EQUAL EQ-5D questionnaires revealed no significant difference in perceived quality of life, including self-reported overall health rating from baseline to the end of the study treatment period, or between treatment groups (Supplementary Table 1).

**TABLE 3:** Effect of the interventions on laboratory blood safety parameters including creatinine kinase, liver function, renal function, fasting glucose, and thyroid function over the 8-week treatment period.

	Placebo		Ezetimibe		Nutraceutical		Combination	
	Week 0	Week 8	Week 0	Week 8	Week 0	Week 8	Week 0	Week 8
Creatinine kinase (U/L)	99 ± 11	103 ± 25	130 ± 20	119 ± 19	110 ± 26	132 ± 30	112 ± 13	120 ± 17
ALT (U/L)	32 ± 4	30 ± 3	31 ± 4	30 ± 3	31 ± 6	46 ± 12	26 ± 4	27 ± 5
GGT (U/L)	43 ± 11	40 ± 11	33 ± 7	30 ± 6	30 ± 9	38 ± 13	27 ± 7	26 ± 4
Glucose (mmol/L)	5.6 ± 0.1	5.4 ± 0.3	6.0 ± 0.3	5.6 ± 0.4	5.7 ± 0.3	5.2 ± 0.2	5.5 ± 0.1	5.2 ± 0.1
Creatinine (μmol/L)	73 ± 5	73 ± 5	78 ± 4	76 ± 3	80 ± 5	76 ± 4	73 ± 5	73 ± 5
eGFR (mL/min)	84 ± 3	83 ± 3	82 ± 2	86 ± 2	80 ± 2	84 ± 3	79 ± 4	78 ± 4
Free T4 (pmol/L)	12 ± 0	12 ± 1	12 ± 0	13 ± 0	12 ± 0	14 ± 1	12 ± 0	14 ± 1
TSH (mU/L)	1.90 ± 0.37	2.14 ± 0.63	1.97 ± 0.28	1.40 ± 0.44	2.34 ± 0.57	3.75 ± 1.59	1.99 ± 0.40	1.77 ± 0.47

Mean ± SEM. ALT, alanine aminotransferase; eGFR, estimated glomerular filtration rate; GGT, gamma-glutamyl transferase; T4, thyroxine, TSH, thyroid stimulating hormone.

## Discussion

The present study has demonstrated the LDL-c lowering benefit of a nutraceutical regimen containing berberine, RYR and plant sterols, alone and in combination with ezetimibe, in patients intolerant to statins who on average were at moderate-to-high cardiovascular risk. The nutraceutical and ezetimibe combination lowered LDL-c by ∼2 mmol/L, and significantly lowered non-HDL-c, REM-c, triglycerides and total cholesterol. The observed reduction in LDL-c with the combination was greater than that seen with either treatment alone. These findings clearly demonstrate the LDL-c lowering efficacy of nutraceuticals in patients with hypercholesterolemia and mixed hyperlipidemia and further demonstrates their practical use as adjunct therapy to other lipid-lowering agents.

There are over 40 nutraceuticals suggested to have lipid-lowering properties and we and others have reported on the benefits of various nutraceuticals, either alone or in combination with ezetimibe (16–19). An International Lipid Expert Panel supports their use as alternative or adjunct therapies in statin intolerant patients due to their relative safety and potential LDL-c lowering properties (20, 21). Armolipid Plus, a proprietary formulation of 6 plant-based compounds has been the most widely studied, with clinical review and meta-analysis revealing significant reductions in LDL-c and triglycerides (22, 23). The nutraceutical regimen in our study consisted of berberine, RYR and plant sterols, which all have distinct yet complementary mechanisms for LDL-c reduction. Berberine acts as a natural PCSK9 inhibitor, the protein responsible for degradation of LDL receptors and thus can increase the number and activity of hepatic LDL receptors (15, 16). Fermentation of RYR by the fungus Monascus purpureus results in the formation of monacolin K (naturally occurring lovastatin), which can inhibit HMG-CoA reductase (15, 16). Several expert opinion position statements suggest that the use of RYR is both safe and effective for reducing LDL-c. While its use shouldn’t be in place of conventional lipid-lowering therapy, it may be considered in specific conditions, including statin intolerance (24, 25). Plant sterols are naturally occurring steroid like substances that are structurally similar to cholesterol, but poorly absorbed in the intestinal tract, thus decreasing the intestinal absorption of cholesterol by lowering cholesterol content in micelles (15, 16). The complementary actions of these three nutraceuticals in the present study resulted in reductions in LDL-c comparable to that seen with ezetimibe, which acts by inhibiting cholesterol absorption *via* the NPC1L1 transporter protein, and importantly, additive effects when taken in combination. Furthermore, this effect was observed without adversely affecting blood safety parameters or the development of self-reported side effects. The beneficial actions of these nutraceuticals may also offer alternative or adjunct therapies to the recently developed bempedoic acid and PCSK9 inhibitors, particularly in patients who are unable to tolerate statins and where use of these therapeutics is restricted. Bempedoic acid has been shown to be well-tolerated and to significantly reduce LDL-c when added to background therapy, including ezetimibe, in both statin-intolerant patients (26, 27), and in high-risk ASCVD and FH patients (28, 29). Analysis of the GAUSS-3 trial showed significant reductions in LDL-c levels in statin-intolerant patients treated with evolocumab compared to ezetimibe, with no significant muscle-related adverse events reported (30).

In spite of these observed positive effects on LDL-c, however, it should be noted that only one patient achieved a LDL-c of ≤ 2.5 mmol/L, the recommended target for moderate risk patients. This may be due in part to the presence of patients with very elevated levels of LDL-c, including 8% of the cohort being classified as having probable phenotypic FH and 28% as having combined hyperlipidemia. FH patients rarely achieve treatment goals with standard therapy. In patients with statin intolerance, treatment with bempedoic acid, bile acid sequestrants or PCSK9 inhibitors would be indicated. However, bempedoic acid is not registered in Australia, none met PCSK9 criteria and bile acid sequestrants are very poorly tolerated owing to gastrointestinal intolerance. As such, although significant reductions in LDL-c were observed, the present study demonstrates the need for further investigation into the benefits of different nutraceutical combinations, or the value of increasing the dose of individual nutraceutical components, particularly in moderate to high-risk and hard to treat patients.

Minor side effects of gastrointestinal upset and myalgia were reported equally across all four treatment groups, including placebo. This may be in part due to the “nocebo” effect; negative expectations about the effects of a treatment, resulting in higher than expected adverse event reporting (31–33). A recent double-blind, three-group, n-of-1 trial demonstrated that in patients who had discontinued statin therapy due to side effects, 90% of the symptom burden caused by a statin re-challenge was also caused by a placebo (34). Large-scale trials have also observed development of SAMS in statin intolerant patients randomized to either PCSK9 inhibitors and/or ezetimibe, drugs that operate *via* mechanisms distinct to statins (35, 36). The true prevalence of statin intolerance is unclear (9, 31, 37, 38), with a recent meta-analysis of randomized controlled trials and observational studies suggesting it is 9.1%, and even lower when using various international guidelines (39). Regardless, non-adherence or discontinuation of statin therapy significantly increases a person’s lifetime risk of major cardiovascular events and is thus a major clinical problem that needs to be addressed (40).

A limitation of the study is that 49% of the cohort were considered to be overweight or obese (BMI > 28 kg/m^2^) and we did not employ a dietary weight loss regimen to assess the incremental effect of weight loss on the primary endpoint of LDL-c reduction. Further, the sample size was relatively small and the period of intervention short. However, statistical power was increased by our factorial design. The formulations used in the study were not strictly practicable but were required to ensure adequate blinding. The strengths of the study were the double-blind, randomized placebo-controlled, factorial interventional design, with close monitoring of treatment related side effects.

In conclusion, the present study has demonstrated the significant beneficial effect of a nutraceutical regimen containing berberine, RYR and plant sterols, alone and in combination with ezetimibe, in lowering LDL-c in patients intolerant to statins. The observed ∼2 mmol/L reduction in LDL-c with the combination therapy is likely to be clinically significant and if sustained, could result in reduction in CVD events (2). Although minor side-effects were reported across all patient groups, the nutraceuticals, either alone or in combination with ezetimibe, had no adverse effects on blood safety parameters or self-reported quality of life. Further research on the formulation of various nutraceutical combinations and doses to improve effectiveness and palatability, whilst still being available at an acceptable cost, is warranted, as well as their use as adjuncts to other treatments, including PCSK9 inhibitors and bempedoic acid.

## Data availability statement

The raw data supporting the conclusions of this article will be made available by the authors, upon reasonable request.

## Ethics statement

The studies involving human participants were reviewed and approved by the Royal Perth Hospital Human Research Ethics Committee. The patients/participants provided their written informed consent to participate in this study.

## Author contributions

NW conducted the study, initial data analysis, and first draft of the manuscript, with subsequent input from CR and GW. All authors contributed to the study design and funding acquisition, reviewed, and edited the manuscript prior to submission.
