# Supplementary material for: Low-density lipoprotein-cholesterol lowering effect of a nutraceutical regimen with or without ezetimibe in hypercholesterolaemic patients with statin intolerance
Source: Front Cardiovasc Med. 2022 Nov 25;9:1060252. doi: 10.3389/fcvm.2022.1060252 (PMC9732015; doi:10.3389/fcvm.2022.1060252)
Supplement: Supplementary file 1 [file Table_1.pdf]

Supplementary Table 1: *Self-reported overall health using the EQUAL EQ-5D quality of life questionnaire completed at weeks 0 and 8.*

	**Placebo**		**Ezetimibe**		**Nutraceutical**		**Combination**	
	**Week 0**	**Week 0**	**Week 8**	**Week 0**	**Week 0**	**Week 8**	**Week 0**	**Week 8**
**Overall health rating /100**	83	84	86	85	86	86	85	84
**Mobility (n)**								
**No problem**	9	11	12	9	12	12	9	11
**Slight problems**	4	2	1	1	1	1	1	2
**Moderate problems**	0	0	0	0	0	0	0	0
**Severe problems**	0	0	0	0	0	0	0	0
**Unable**	0	0	0	0	0	0	0	0
**Personal care (n)**								
**No problems**	10	13	13	9	13	13	9	13
**Slight problems**	3	0	0	1	0	0	1	0
**Moderate problems**	0	0	0	0	0	0	0	0
**Severe problems**	0	0	0	0	0	0	0	0
**Unable**	0	0	0	0	0	0	0	0
**Usual activity (n)**								
**No problems**	10	12	13	9	13	13	9	12
**Slight problems**	3	1	0	1	1	0	1	1
**Moderate problems**	0	0	0	0	0	0	0	0
**Severe problems**	0	0	0	0	0	0	0	0

**Unable**	0	0	0	0	0	0	0	0
**Pain / discomfit (n)**								
**No problems**	2	8	7	5	4	7	5	8
**Slight problems**	6	4	4	3	8	4	3	4
**Moderate problems**	5	0	2	1	1	2	1	0
**Severe problems**	0	1	0	1	0	0	1	1
**Unable**	0	0	0	0	0	0	0	0
**Anxiety / depression (n)**								
**No problems**	10	8	11	7	9	11	7	8
**Slight problems**	2	3	2	3	3	2	3	3
**Moderate problems**	0	1	0	0	1	0	0	1
**Severe problems**	1	1	0	0	0	0	0	1
**Unable**	0	0	0	0	0	0	0	0

No significant difference between week 0 and week 8 or treatment groups for any parameters.
